# Assessing competence needs for doctors in the emergency department duty rosters: an observational study

**DOI:** 10.1186/s12245-023-00515-y

**Published:** 2023-06-20

**Authors:** Johannes Kolnes, Erlend Hodneland, Audun Lange, Torhild Heggestad

**Affiliations:** Department of Research and Development, Haukeland University Hospital, Bergen, Norway

**Keywords:** Emergency epidemiology, Duty roster, Competence requirements, Frequency distribution, Exponential pattern

## Abstract

**Background:**

The purpose of our investigation is to analyze if emergency epidemiology is randomly variable or predictable. If emergency admissions show a predictable pattern, we can use it for multiple planning purposes, especially defining competence needs for duty roster personnel.

**Method:**

An observational study of consecutive emergency admissions at Haukeland University Hospital in Bergen over six years. We extracted the discharge diagnoses from our electronic patient record and sorted the patients by diagnoses and frequency. Data were loaded into a Jupyter notebook and presented in form of frequency diagrams.

The study population, 213,801 patients, comprises all emergency admissions in need of secondary emergency care from the relevant specialities in the catchment area of our hospital in the western health region of Norway. Patients in need of tertiary care from the whole region are also included.

**Results:**

Our analysis shows an annually reproducible distribution pattern regarding type and number of patients. The pattern adhere to an exponential curve that is stable from year to year. An exponential distribution pattern also applies when we sort patients according to the capital letters groups in the ICD 10 system. The same applies if patients are sorted adhering to primarily surgical or medical diagnoses.

**Conclusion:**

Analysis of the emergency epidemiology of all admitted emergency patients in a defined geographical area gives a solid basis for defining competence needs for duty roster work.

**Supplementary Information:**

The online version contains supplementary material available at 10.1186/s12245-023-00515-y.

## Background

One of the important dilemmas in hospital medicine today is to achieve a balance between specialised diagnostic and therapeutic competence needed in the daily, subspecialised practices, and the general diagnostic and therapeutic knowledge needed for duty roster service 24/7. Modern medicine requires increasing levels of specialisation [1, 2]. Once a diagnosis is established, we are all in favour of getting our treatment from the best specialists available. However, 2/3rd of patients admitted to general hospitals are emergencies. Many of them do not have an obvious attachment to a medical speciality or a clear diagnosis [3]. All patients that are misdiagnosed experience unnecessary hassle, poor communication and broken trust. Worse though is it that misdiagnosis or delayed diagnosis often lead to poorer prognosis for the patients through prolonged illness, increased temporary or permanent disabilities or even death. Allocating optimal medical competence to ensure correct diagnosis, is therefore a decisive challenge. An evaluation of the emergency services in the Netherlands has shown that breaking down emergency services into standardised components and subsequently improving one component after another is a practical way to systematically improve emergency services [4]. Our focus is on the diagnostic process, primarily the competence spectre of duty roster doctors They will have to obtain a general knowledge of emergency medicine procedures. Furthermore they should know how to diagnose and treat the diseases constituting the local emergency epidemiology. The aim of our study is to analyze if emergency epidemiology is randomly variable or predictable. If emergency admissions show a predictable pattern, we can use it for a range of planning purposes, e.g., type and capacity of diagnostic equipment, emergency ward bed capacities, emergency personnel requirements etc. We also want to show that it is suitable to define the spectre of diagnostic and therapeutic competence needed for local duty roster personnel.

Our hypothesis is that it is possible to establish a local, comprehensive list of emergency diagnoses, the frequency of their occurrence and their frequency relative to each other in spite of their random appearance.

## Methods

Our observational study on emergency admissions is based on data from Haukeland University Hospital. It is a public hospital delivering secondary and tertiary care in the western health region of Norway and caters for 315,000 (2020) inhabitants as a local hospital and a population of 1.1 million as a tertiary facility. The Emergency Clinic receives patients for acute admission for all emergencies except psychiatric, paediatric, gynaecological and obstetric patients. The doctors on call on the duty rosters in the Emergency clinic are partly employed in various medical and surgical departments and partly the Emergency departments own doctors.

The Norwegian primary health care doctors are gatekeepers before hospital admissions. Still, even in central areas like ours, 52% of emergency admissions are self-referrals, patients arriving without any prior medical professional assessment [5]. This underlines the need for a competent emergency diagnostic capacity in our hospital. The public health care system is the sole supplier of emergency care in Norway. The accumulation of medical information on emergency patients starts prehospitally by primary health care staff and ambulance personnel. The data registered are personal information (ID), sex, age, the tentative admission diagnosis, and other relevant information available (high/low energy trauma, other injury mechanisms, time of onset of symptoms, type of symptoms etc.) After arrival in the hospital, we manually transfer the relevant prehospital data to our Electronic Patient Record (EPR). The patient is triaged on arrival by the triage nurse. Then, the on-call doctor does a clinical examination. Afterwards/simultaneously this is supplemented with, biomedical tests, and X rays. Subsequently we conclude on the admission diagnosis as basis for the emergency treatment. The patient is then affiliation to a specialized ward and transferred for further observation and treatment. Here, we continue treatment until discharged. The doctor responsible for the treatment documents the final diagnosis in our EPR on discharge. We have used the main discharge diagnosis in our analyses.

We have done an observational study of all emergency admissions through our Emergency Clinic from 2015 to 2020. Our material makes up all emergency admissions in our catchment area for patients admitted to the Surgical clinic (all subspecialties), the Medical clinic (all internal medicine specialities), the Orthopaedic clinic, the Cardiology clinic, the Neurological clinic (including Neurosurgery), the Lung disease clinic, the Head and Neck clinic and the Emergency clinic.

The total number of emergency admissions to these clinics over the period of 6 years was 213,801. Patients discharged with a diagnosis from the Z group (ICD 10) were excluded as these patients by definition are non-emergencies. There were 5778 admissions in this category. No other patients in the defined cohort were excluded.

### Patient and public involvement

We invited two members of the hospitals’ permanent user forum into the research group at the start of the project. The plenary group had three meetings. Later, during the writing process, we gave the user representatives equal opportunity to amend and comment on the text. Initially, they have found it difficult to build opinions on the subject under debate as they found that it required some general medical knowledge. They only had that knowledge on the diseases they knew of from their patient unions; hence, the cross sectional knowledge was lacking. However, when focusing on the general managerial aspects’ logistics, staffing, delays etc. they felt that they were able and competent. This had minor influence on the project.

### Statistical analyses

In Norway, we report information on all hospitalised patients to a national database, the National Patient Register (NPR). The transferral xml-format, named the NPR-record, was used to extract patient information. Structured table data of emergency admissions to our hospital with individual records of ICD-10 code of main discharge diagnosis, ICD-10 chapter, and year of hospitalization were loaded into a Jupyter notebook (http://Jupyter.org). An important detail to achieve the right level of detail for such an analysis is to omitt the last cifre of the ICD 10 code as this cifre just describes details like where in the breast a breast cancer is located. 

We listed diagnoses according to their individual ICD-10 chapter names, with chapters A and B combined into one group, while other chapters (e.g., C, D) remained individual entities. The table reached 213,801 individual emergency hospital admissions from 2015 until 2020. For each year, we counted the number of occurrences of diagnoses within individual ICD-10 chapters, and sorted histograms showing the sample mean and 95% prediction interval (PI) of diagnoses across the reported timespan.

We fitted an exponential decay ${f\left(x\right)={b}_{0}e}^{-{b}_{1}x}$ to the histogram of diagnoses using scipy.optimize (https://scipy.org) with initial values ${b}_{0}=1000, {b}_{1}=0.1$. We also fitted a Pareto distribution of diagnoses using the power law representation

$$f\left(x|\alpha ,k,C\right)=C\frac{\alpha {k}^{\alpha }}{{x}^{\alpha +1}}, k\le x<\infty ; \alpha ,k>0.$$

Optimal $\widehat{\alpha }$ has the following form using the method of maximum likelihood [6]

$$\widehat{\alpha }=n/\sum_{i=1}^{n}log\left(\frac{{x}_{i}}{\widehat{k}}\right)$$

given the sample data ${x}_{i}$ with $n$ elements and $\widehat{k}=\mathrm{min}\left({x}_{i}\right)$, since $x$ can never be smaller than $k$.

Admitting a patient is a subjective process based on assessment of severity of disease. This result in a potential selection bias underestimating the number of cases due to omittance of less severe cases treated as day- or outpatients.

The TRIPOD guidelines for transparently reporting multivariable prediction models for individual prognosis or diagnosis, was used (Collins GS, Reitsma JB, Altman DG, Moons KG).

## Results

**Fig. 1:**
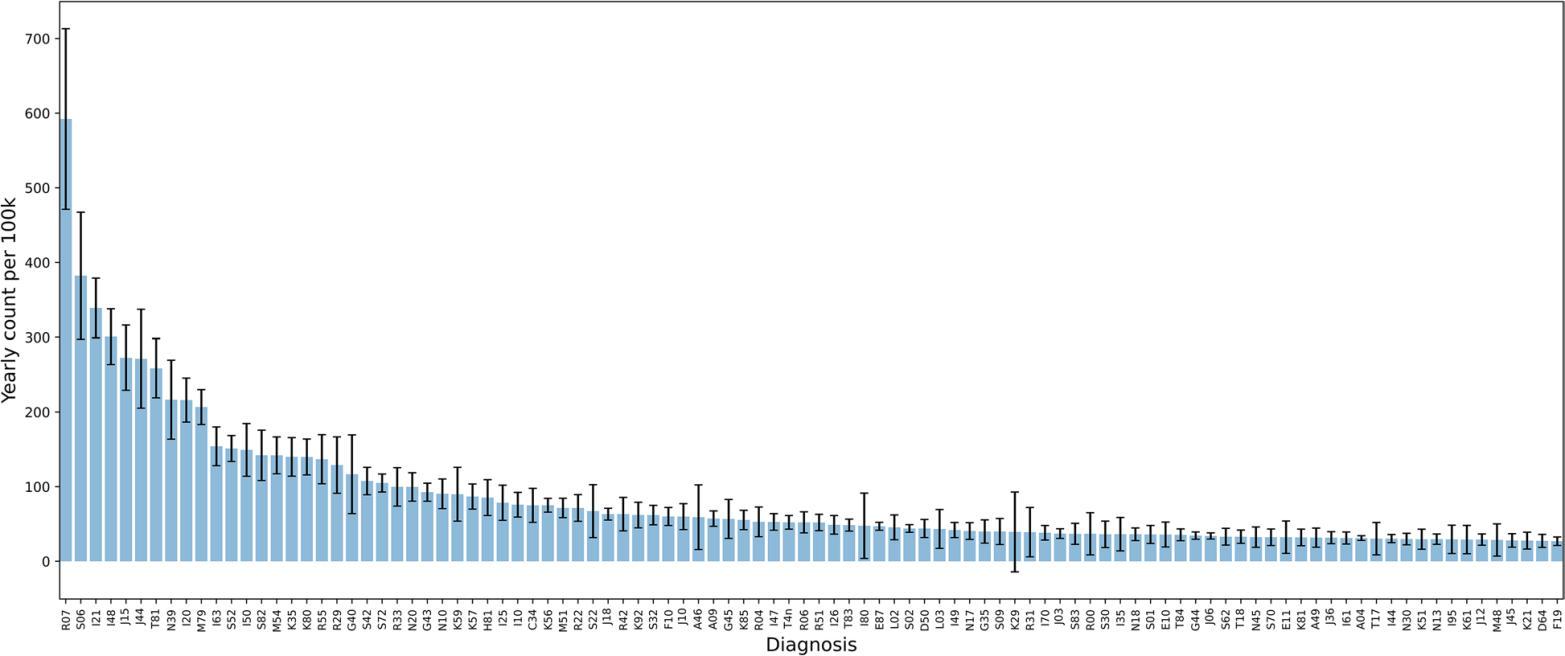
Diagnoses according to ICD 10. The average count of diagnoses making up 80% of admissions 2015–2020 with 95% prediction intervals

Figure 1 shows the frequency diagram with the prediction intervals for the diagnoses comprising 80% of patients. To avoid the long tail of rarer cases we have omitted the least frequent 20% diagnoses.

The histogram reports normalized frequencies relative to 100,000 (100 k) inhabitants. Our compilation of data clearly shows that there is a reproducible pattern in the distribution of acute illness. There is a surprising consistency over the years in spite of the inherent stochastic variation of arrival in the ER. The histogram has an exponential distribution. The variation of the measurements for the different diagnoses over the 5 years show relatively minor fluctuations. This does not affect the distribution sequence of diagnoses significantly year by year. Neither does the general increase in emergency admissions over the years affect the distribution sequence.

**Fig. 2:**
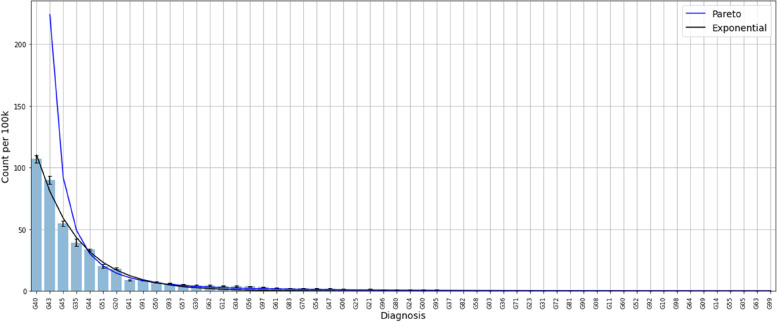
Distribution of diagnoses in the G group according to ICD 10, Neurological diseases. We fitted an exponential and a Pareto model to the histogram, mostly suggesting an exponential decay for the diagnoses

Subsequently, we did the same kind of analysis for all patients in each capital letter group of ICD10. All of the analysis are available in Supplement A. To give some examples, Fig. 2 shows the distribution of Neurological diseases, the ICD 10 group G.

The distribution of acute neurological diseases illustrate a typical Pareto distribution as used by M. King in the WHO term Community Diagnosis [7]. From a mathematical point of view it follows an exponential curve better than the theoretical Pareto distribution as illustrated.

**Fig. 3:**
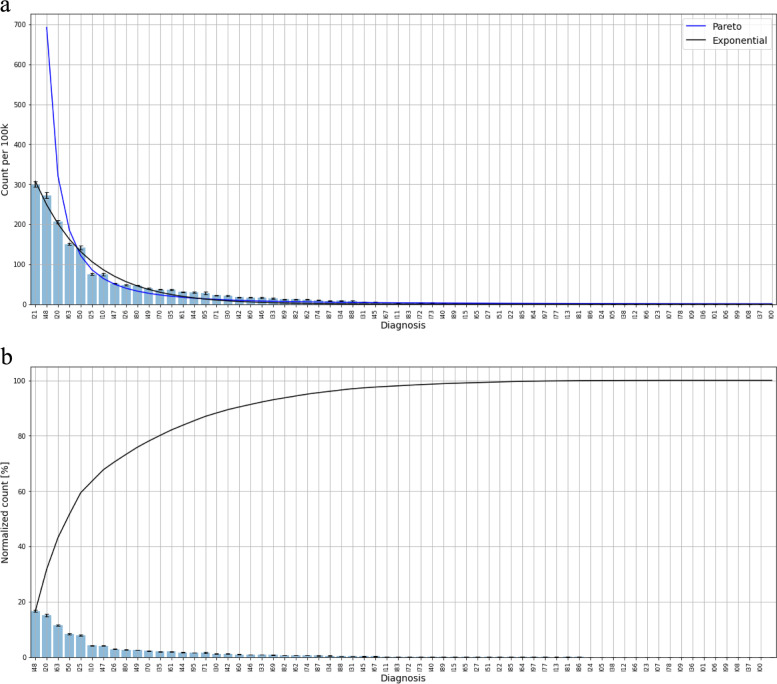
**a** Distribution of diagnoses in the I group, cardiovascular diseases. The data adhere to an exponential fit. **b** Cumulative curve of I diagnoses in the I group, cardiovascular diseases

In the I group, cardiovascular disease, Fig. 3a, there is an exponential distribution. The ten most common diseases comprise 75% of the total best defined by using the cummulative curve in Fig. 3b. There is a tendency to have minor variation in the sequences in the frequency diagram from year to year, but the number of patients remain predictable. Overall, the selection is still pretty stable. Some of the largest diagnostic groups according to the ICD 10 chapter of I, are attributed to several medical specialities. Acute coronary infarction and atrial fibrillation belongs to cardiology. The third largest group, I63 brain stroke, belongs to Neurology. The cummulative curve in Fig. 3b allows easy identification of the diagnoses comprising 80% og the total number of patients in the I group. This curve can be used to identify the most common diseases up to a desired level also in the other main groups of diagnoses (capital letter groups).

**Fig. 4:**
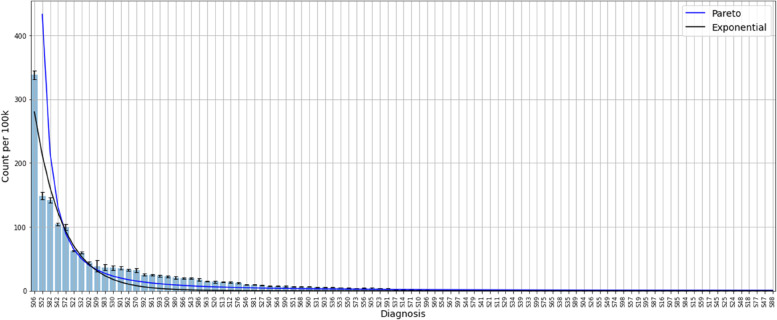
Distribution of diagnoses in the S group, Injuries and accidents

The S group in Fig. 4 shows an example of a more flattend distribution, close to accordance with Power law distribution, where the ten most common injuries still covers a clear majority of cases but does not, at 66%, reach the 80% limit qualifying for a pure Pareto distribution as defined by King [7]. The stability over the years still is surprisingly coherent given that these diagnoses predominantly are caused by accidents and injuries. Others have previously shown that accidents and injurie happen far from random [8].

**Fig. 5:**
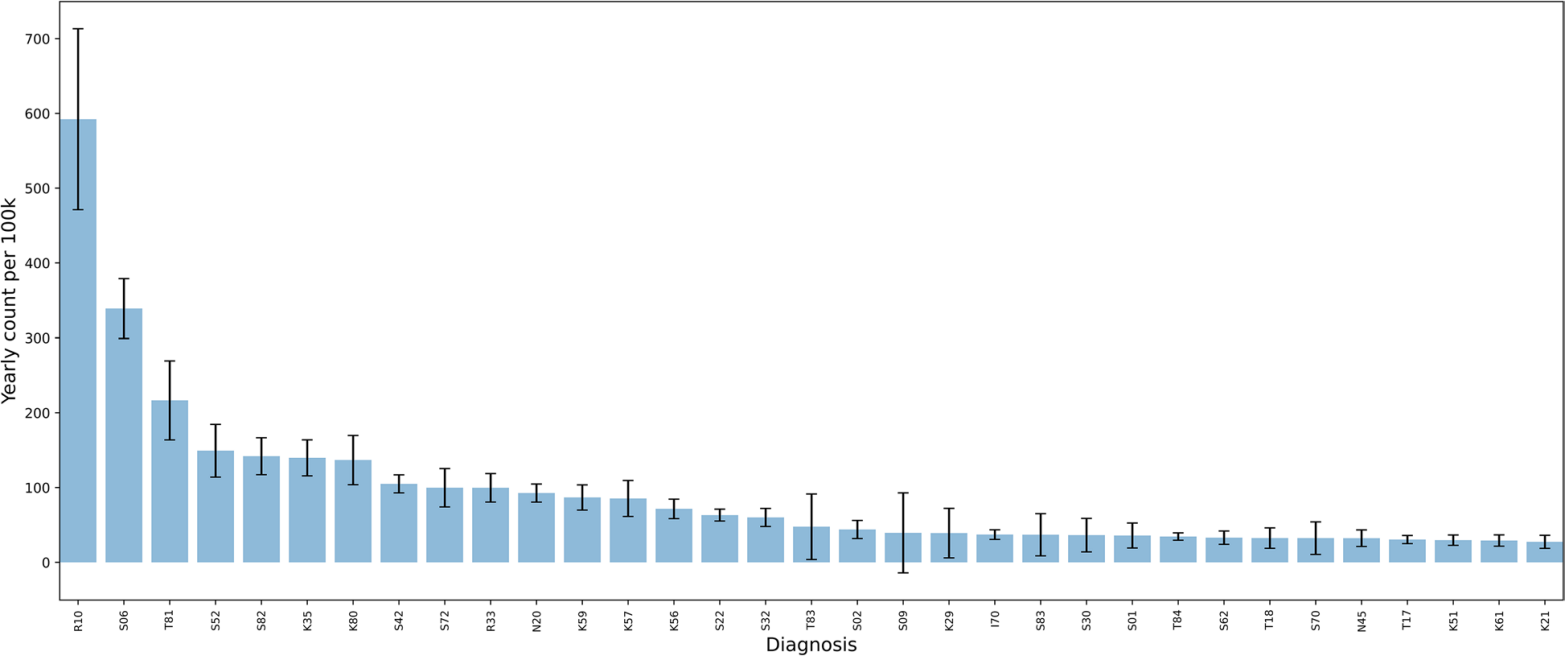
Surgical diagnoses

**Fig. 6:**
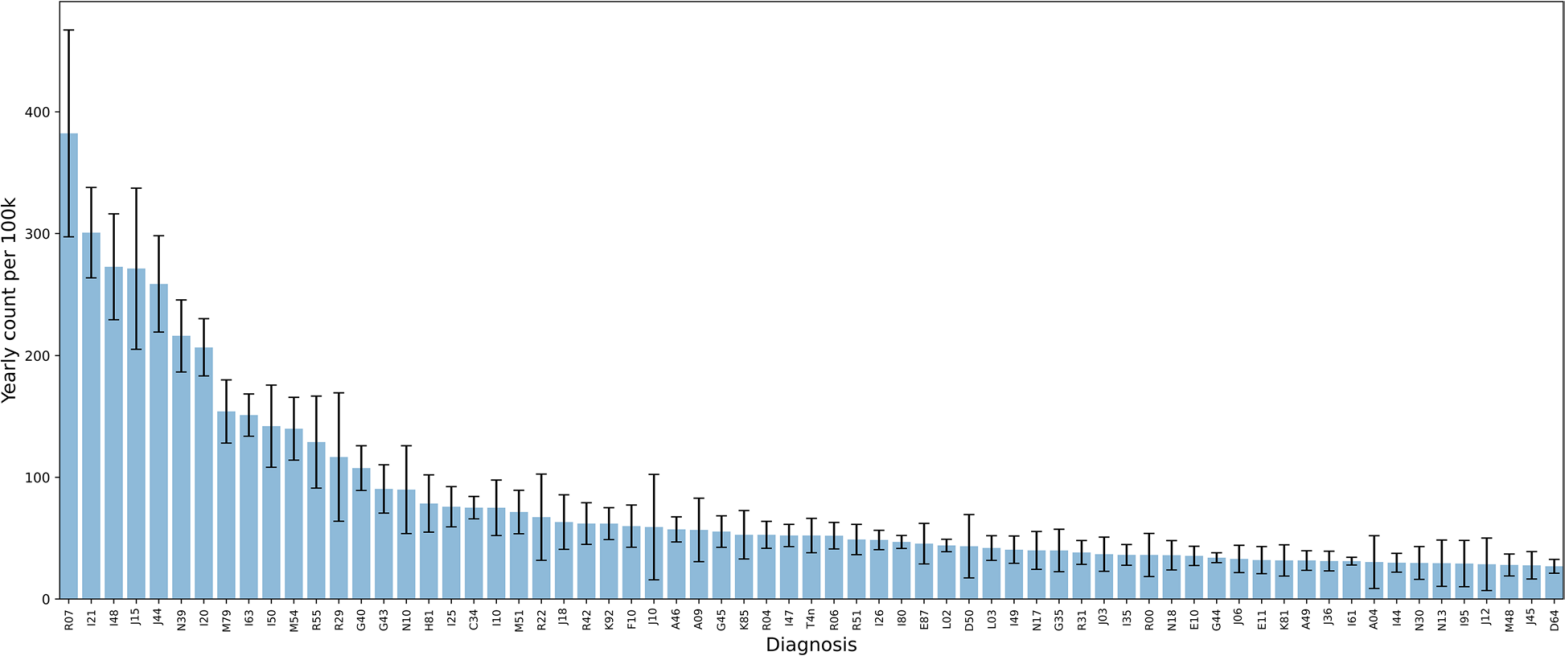
Medical diagnoses (non-surgical)

Building the competence for duty roster personell represent an important challenge. A third way of analysing the diagnoses in the frequency diagram is to arrange them according to type of duty rosters locally. Most small and medium sized hospitals have one group of doctors covering the surgical patients and another group covering the medical patients. Hence, we have split the frequency diagram in two of which one contains surgical diagnoses (Fig. 5), and another contains the medical diagnoses (Fig. 6). The distribution in these diagrams reflects the patient distribution routines of our hospital. Other hospitals will have slightly different distributions according to their local policies.

## Discussion

To our knowledge, clinically useful analyses of the total distribution of diseases from the hospital catchment area or local communities, are rarely published The literature offers abundantly analyses covering selected illnesses as exemplified in the references [9], Urological emergencies, [10], myocardial infarction, and [11] sports injuries. Others do give the overall picture, but with a bird’ eye view perspective which is lacking sufficient detail for clinical use, e.g., national level analysis [12]. Another problem in the literature is that the organisation of health care, e.g., mix of private and public hospitals serving one community, prevents one of these hospitals to represent all emergencies in that community [6]. We have not found other observations covering hospital emergency admissions over several years.

Our material represent all emergency admissions in the above mentioned specialities in our catchment area for six consecutive years. Part of our empirical observation is of a general character in the sense that it can be used in any emergency hospital. However, one must bear in mind that the strength of this kind of observation is that it is tailored to the defined catchment area based on local factors. Such factors might be climatic (hot, cold, wet or dry climate), demographic (ethnicity, genetic-, age-, and sex-distribution of population), occupational (maritime, agricultural, industrial, or office based businesses), or socioeconomic factors. Such variations can be exemplified by the prevalence of parasitic diseases in hot climate (malaria, schistosomiasis), local clusters of genetic disease (porphyria, Chorea Huntington), or increased prevalence of infectious diseases in deprived communities. Such variations will influence the way one judges symptoms in the emergency diagnostic process significantly. The existing literature offers a patchy epidemiological map consisting of prevalence and incidence of single diagnosis or smaller groups of diagnosis from different parts of the world, in our examples from Turkey, South Africa, Italy, and USA. The main gap in the existing literature is that on this account, it will be inaccurate and insufficient for the casual reader. There are two reasons for this. Firstly, because it does not fit the readers environment. Secondly, that it does not cover the majority of diseases in that community.

This paper describes a method available to anybody with access to an electronic patient record to make their own analysis fitted to their own catchment area thus getting a solid basis for a whole range of health or hospital planning purposes both relating to prevention, curative, and rehabilitative medicine.

Possible reasons for the distribution of admissions for acute illness seeming to adhere to exponential patterns, are unknown. Interestingly, power law relations are frequent in biology and observed for entirely different processes with various etiology [13], e.g., the frequency of use of words in any human language, the numbers of species in biological taxa. Modelling the volume of acute illness using exponential distribution can help predict future incidence of acute illness with a high accuracy. Applying a smooth model function instead of short-ranged, empirical data sets for predicting future behavior of disease distribution has an advantage of being less sensitive to random variation in the data. To our knowledge, the finding that disease distribution pattern of emergency cases adhere to power- or exponential law distributions, has not been proven before. This analogue to other areas of biology might indicate that the exponential distribution reflects some sort of a law of nature caused by local factors affecting disease development and the distribution patterns of diseases. Another concequence could be to search for common pathophysiological mechanisms among different types of disease. Further research to confirm such a finding will be needed.

Our observation shows that the distribution of acute illness seems to follow an exponential pattern also for each of the ICD 10 chapters. Some of them even qualify for the the pattern of Power laws [14, 15] revealing the “vital few” diagnoses of the corresponding diagnostic group. The notion “vital few” refers to the few diseases making up a dominant majority of the case load. This kind of analyses was originally used by WHO in analysing the disease burdon in local communities in developing countries under the term Community diagnosis [12]. Later this approach has been used by the WHO in the Healthy City Project [16, 17]. In local communities the ten most common diseases will mostly make up > 80% of the admissions/visits. We have shown that analysed speciality by speciality this rule of thumb applies to even larger population samples. When we, on the other hand, analyze the total burden of emergency illnesses in larger populations, we need far more than 10 diagnoses to reach 80% of patient load. Still the relevant diagnoses and their annual admission incidences and sequence are stable and reproducable.

The notion “Vital few” refers to priority and sustainability. To achieve sustainability one has to give priority to high volume cases and streamline their treatment maximally. Especially, a smooth cross-professional team function is essential for the common cases allowing for optimalization of resource allocation through delegation and task sharing among professions. There should be minimal difference in how the various doctors handle standard cases. Otherwise, delegations will function poorly as no common routine prevails. High variability will thus result in poorer quality of care.

Smooth handling of volume cases will allow more time and resources to the rarer cases as and when they appear. Optimization of rarer cases is more difficult as routine maintenance is more challenging.

The analysis of all emergency diagnoses equals to a large part the spectrum of diagnostic competence required in Emergency Medicine. Alternatively, in hospitals without emergency medicine specialists, it comprises the competence requirements for duty roster personnel. However, one must bear in mind that emergency epidemiology and volume cases only forms the basis. It does not substitute for an additional knowledge of critical emergency procedures with relevance across the diagnostic spectre [18]. Rarer cases are identified and can be added to the education plan if need be, e.g., if they score high on severity or often result in permanent disabilities.

The doctors on the duty rosters come from various surgical and medical specialities. In their daily practice, they cater for a narrow range of patients within their own speciality. In the emergency room, they must be prepared to diagnose and treat a random and much broader range of cases than they see on day duty. The analysis of emergency admission diagnoses gives a clear picture of the “common denominator” across relevant specialities of diagnostic and therapeutic competences that are required for on call services in the emergency department. In small- and medium-sized hospitals the duty roster is divided between predominantly medical or surgical competencies resulting in two on call teams, a medical and a surgical team. Having an oversight over the possible diagnosis one might encounter is considered advantageous as it gives focus for update on new developments in disease mechanisms, diagnostic improvements, and updated treatment options. In addition, it improves work environment factors by reducing uncertainty and stress. Being updated on developments in emergency medicine requires focus and priority. A list of diagnoses to be encountered locally is an important tool to stay on top of this challenge.

Figure 5 shows that there are relatively few diagnoses in the surgical spectre.

Figure 6 shows medical diagnoses. These are approximately double the number of surgical diagnoses.

To guide ongoing training and competence maintenance for all cases, one can use other criteria than high volume. High risk of fatal outcome, high risk of permanent disability, high complexity, high cost etc. are all relevant focuses that also will cover rarer types of diseases. For periods, one can use such priorities for the planning of training. However, one should periodically return to the maintenance of competence on high volume cases.

The minimum level of competence the duty roster personnel must cover is the high-volume cases (80%). Back-up specialists can contribute on the last 20% of rare cases and help advice on the difficult cases in the high frequency group.

The analysis of the individual ICD 10 chapters offers important information to competence building in various medical specialities. Which are the “bread and butter” competencies covering “the vital few diagnoses”, the > 80% majority, and which types of patients are rarely seen.

### Strengths and weaknesses

The major strenght of our analysis is that it is based on a stable population cohort over several years. Furthermore, the organisation of Norwegian health care ensures that the public hospital will cover all emergency admissions in the catchment area. This gives the complete picture and reduces selection bias significantly.

A weakness of our study is the exclusion of multi-morbidity. Regardless of the definition, multi-morbidity is a huge and growing problem [19]. We believe that our principal approach can address this issue in an equally systematic manner.

The discrepancies between the admission and the discharge diagnosis is a further weakness. Analysing the spectre of admission diagnoses behind each discharge diagnoses will make a sound supplement to build accurate diagnostic algorithms. That is beyond the scope of this paper.

The involvement of patient representatives has been challenging. Continuity over several years with turnover and health issues can be challenging. The users felt that they lacked the general medical knowledge required. We tried to convey a message that general administrative knowledge, like assessment of quantitative needs, competence needs for the professionals etc. could be factors where we wanted their opinions. Based on this input, they felt that they could participate in priority of challenges, assessment of suggested solutions formulating text etc. We enclose a summary comment from the user representatives in the Supplementary material B.

Our methodology include readmissions. This reflects the actual workload better than a pure epidemiological picture. Accordingly, the results must not be confused with the annual incidence of specific diseases.

This kind of analysis was very difficult to perform one or two decades ago. With the emerging use of electronic health records both in primary and secondary care, this kind of analysis is easily achievable in the majority of hospitals and practices.

## Summary

- We have conducted an observational study of all emergency admissions in a defined cohort
- The study covers a continuous duration of 6 years
- Frequency diagrams reflecting the observations shows an annually repetitive pattern of disease distribution
- The disease patterns are useful for many sorts of planning purposes

## Conclusion

This paper shows how thorough analysis of local epidemiology proves to be an important tool in planning of emergency services, by systematically defining the common competence requirements for duty roster doctors from various medical subspecialties.

## Supplementary Information

**Additional file 1: Supplementary material A.** Distribution patterns of all Emergency diagnoses. **Supplementary material B.** User representative statement.

## Data Availability

All data generated or analyzed during this study are included in this published article and its supplemented information files.
