# Supplementary material for: Assessing competence needs for doctors in the emergency department duty rosters: an observational study
Source: Int J Emerg Med. 2023 Jun 20;16:39. doi: 10.1186/s12245-023-00515-y (PMC10280889; doi:10.1186/s12245-023-00515-y)
Supplement: Supplementary file 1 — Additional file 1: Supplementary material A. Distribution patterns of all Emergency diagnoses. Supplementary material B. User representative statement. [file 12245_2023_515_MOESM1_ESM.docx]

1. **Supplementary material:**

Distribution patterns of all Emergency diagnoses:

Group A and B


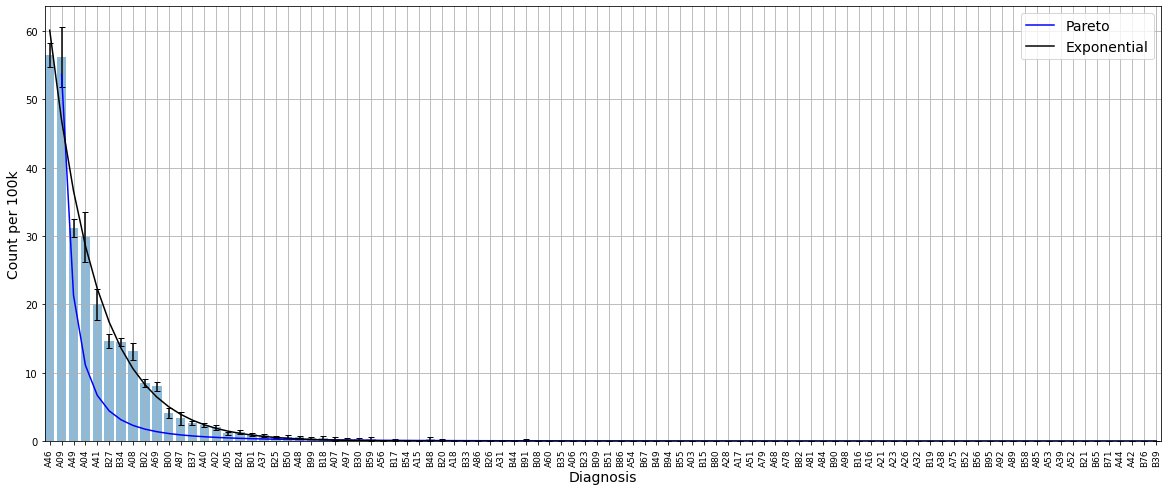


Group C


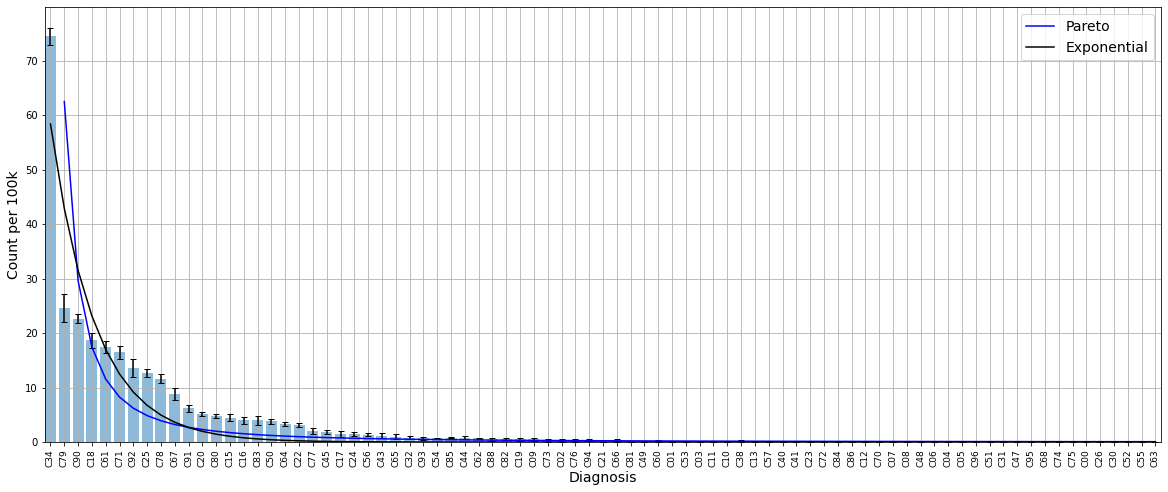


Group D


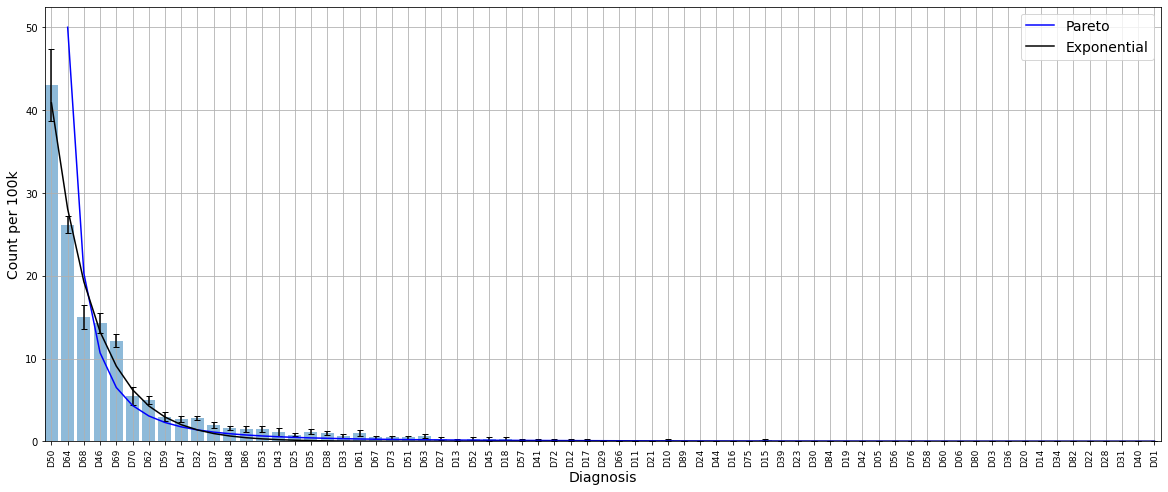


Group E


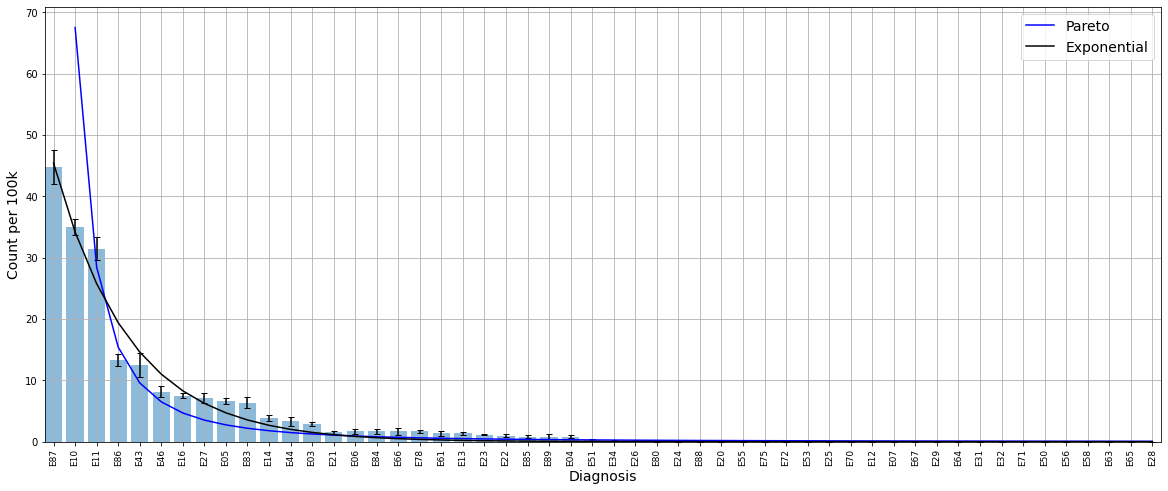


Group F


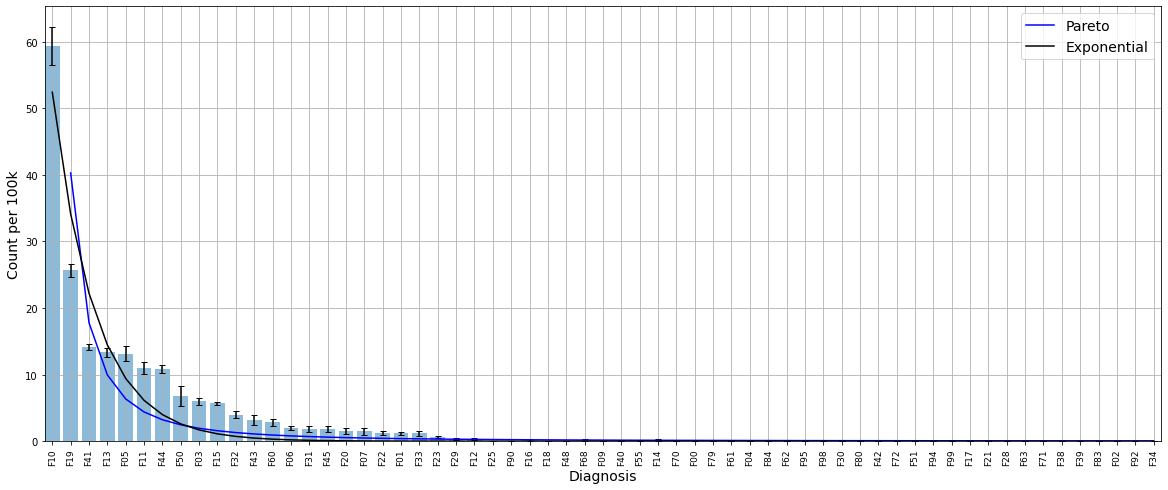


Group G


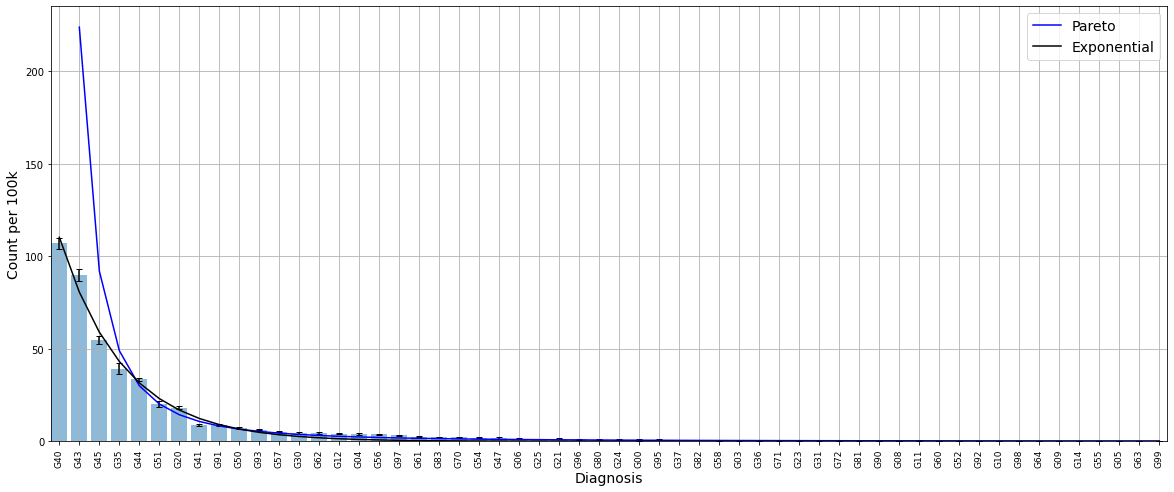


Group H


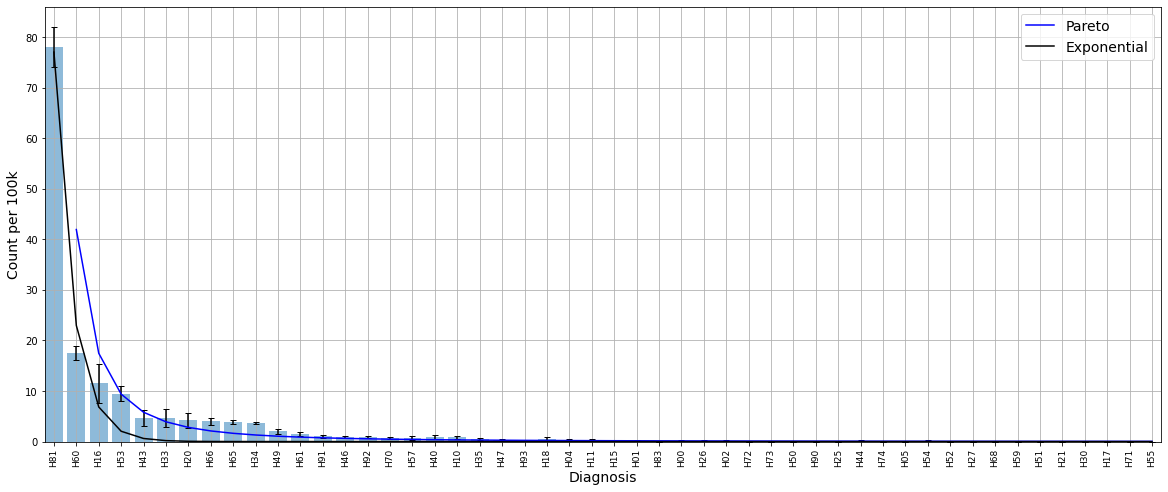


Group I


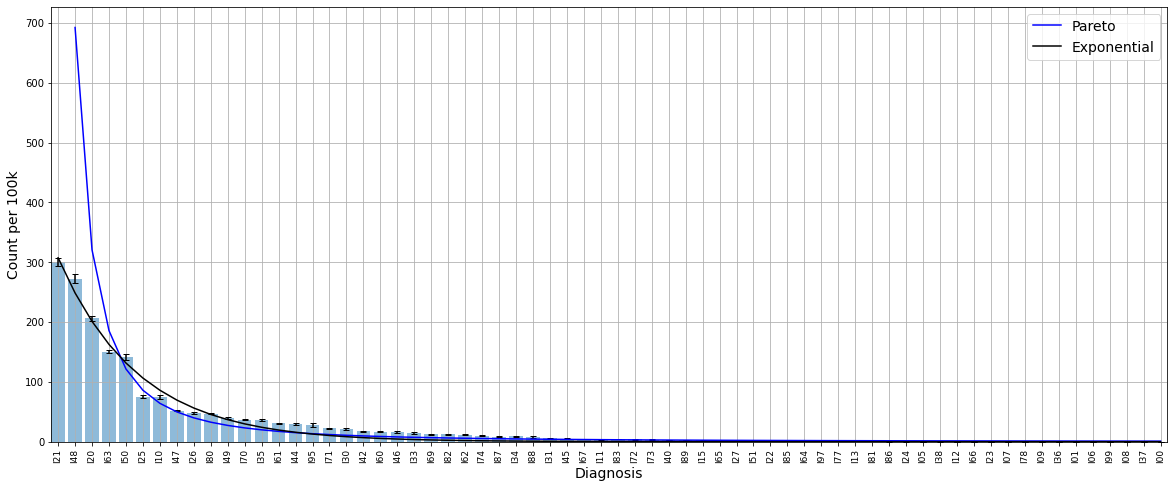


Group J


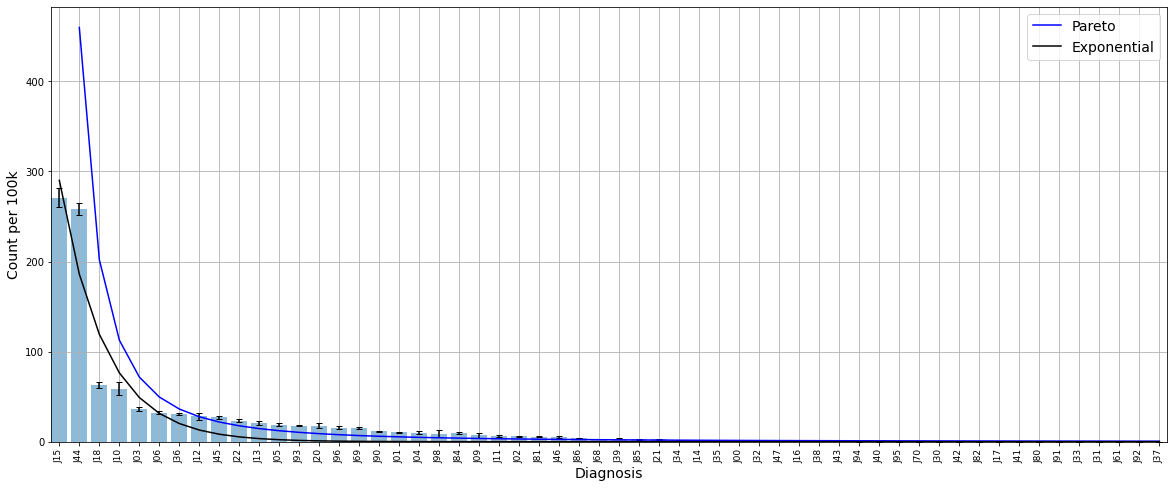


Group K


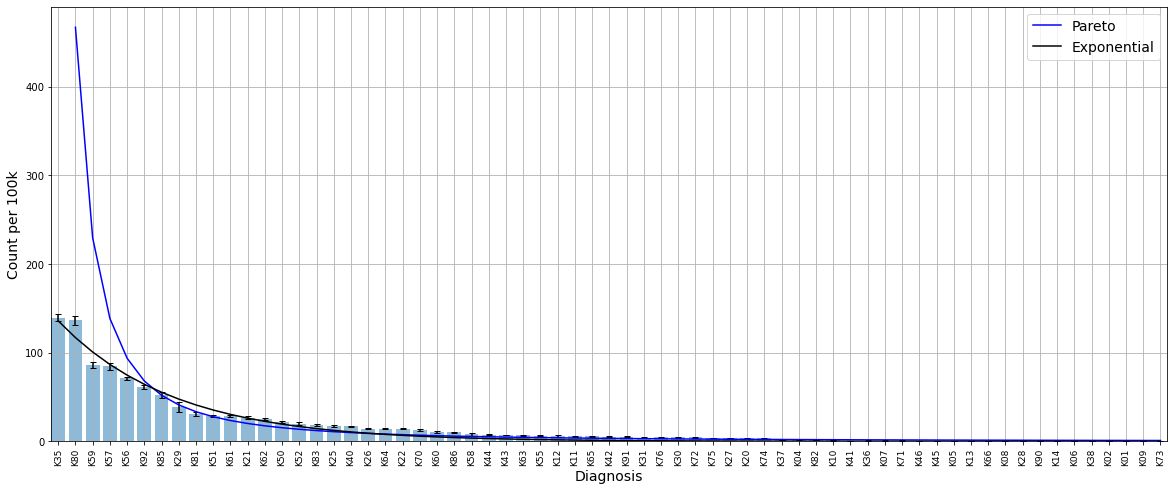


Group L


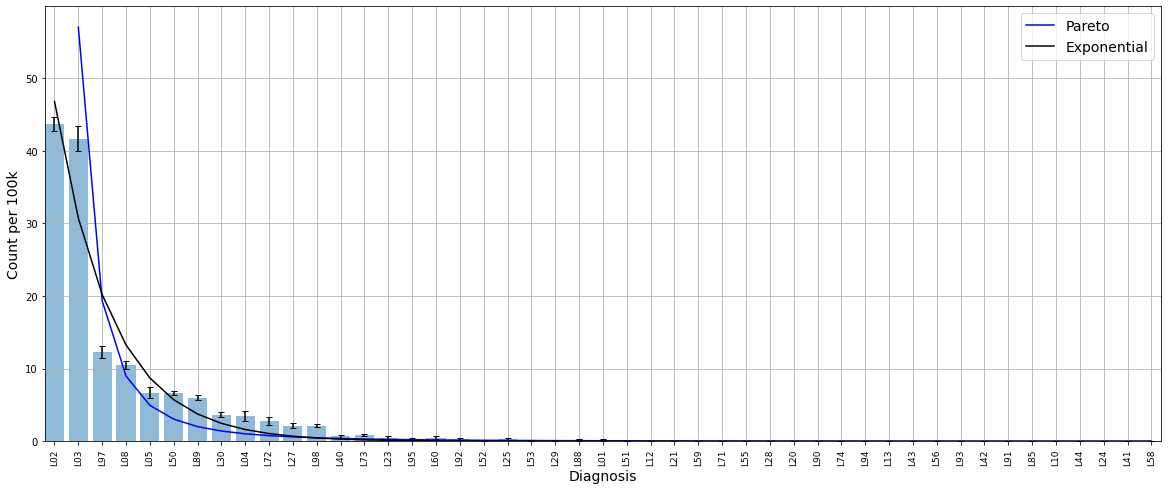


Group M


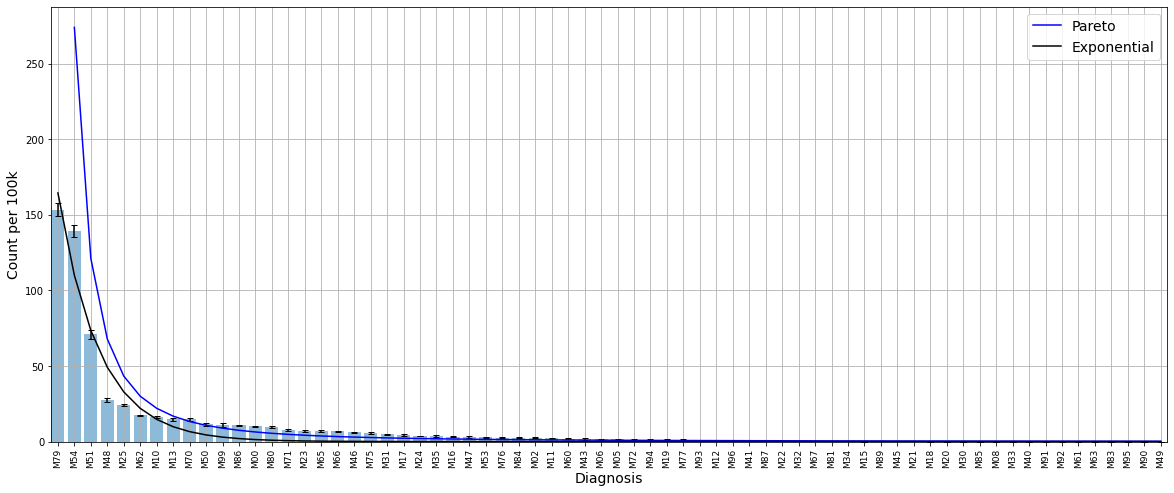


Group N


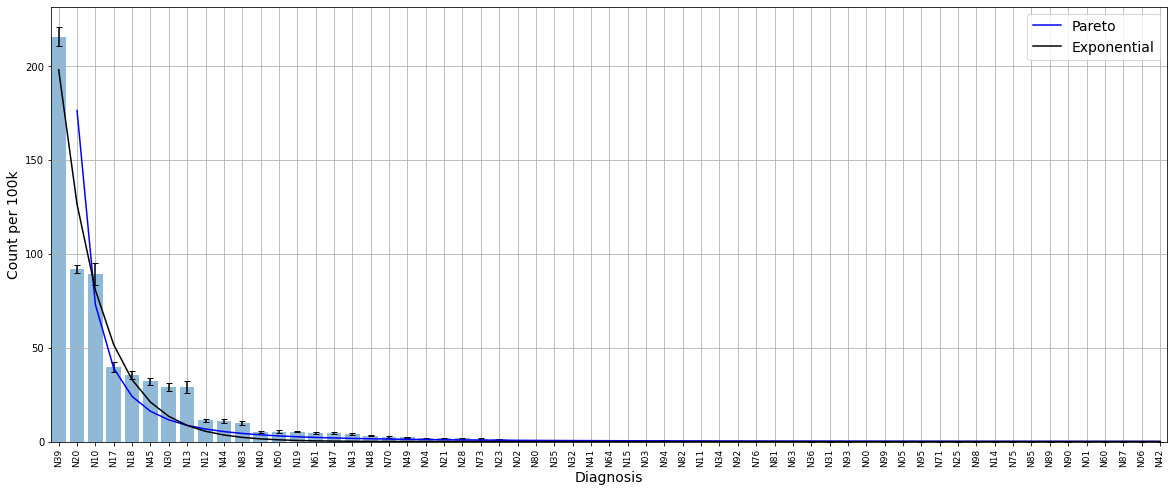


Group R


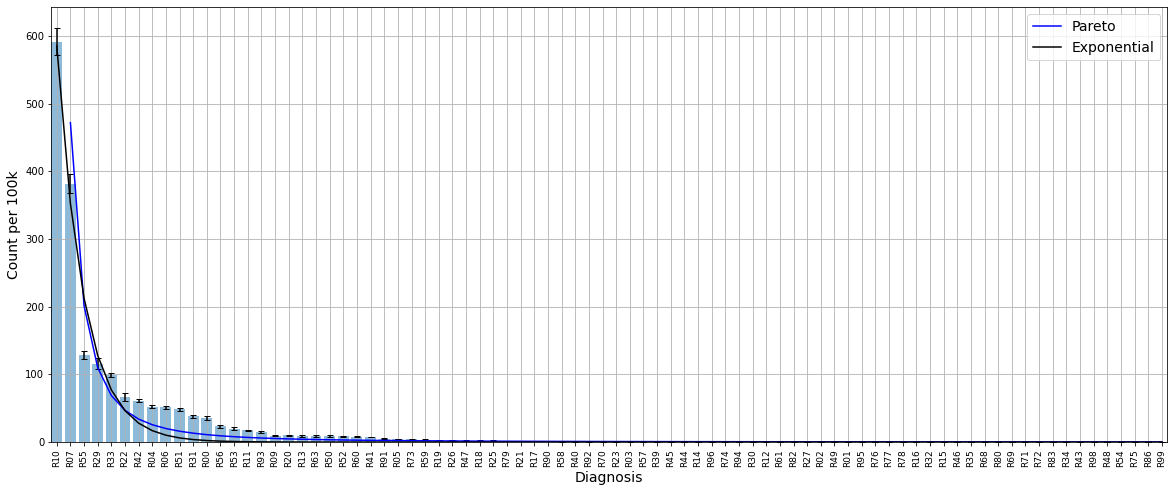


Group S


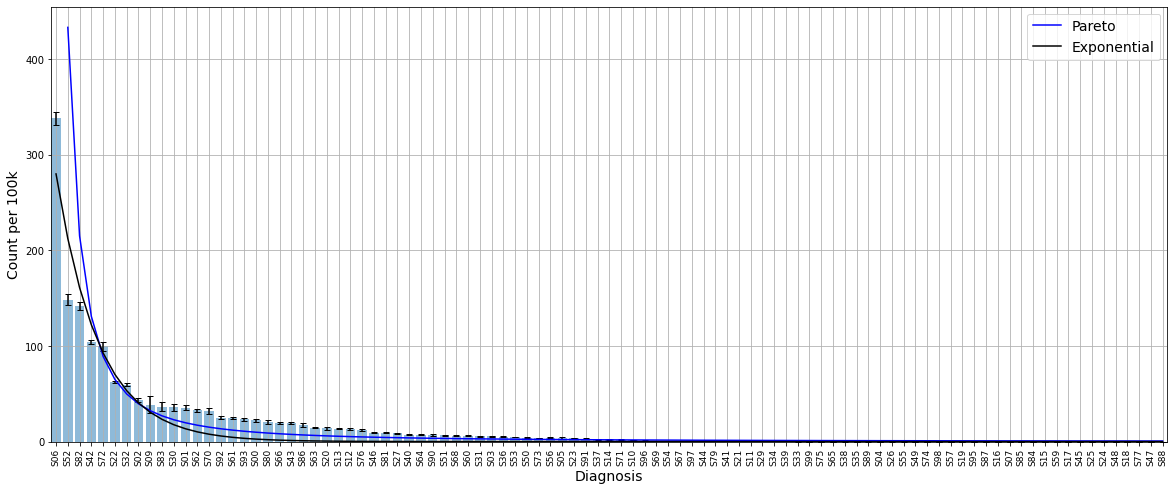


Group T


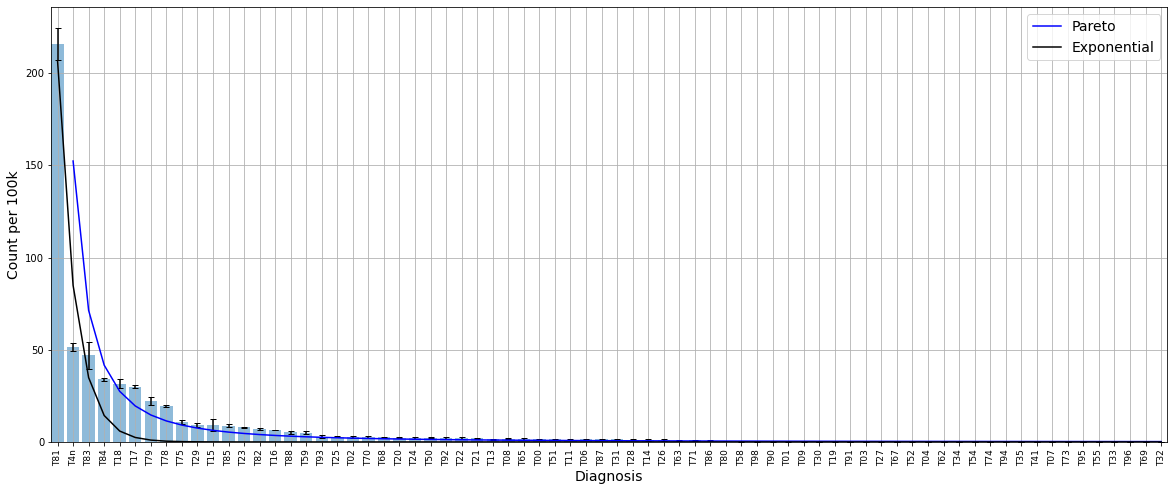


With reference to the department attached to the emergency department we have omitted figures from the groups O,P,Q as these emergencies are treated in the Pediatric -, and Womans Health Clinics. The numbers in our material are negligible.

1. **Supplementary material:**

User representative statement:

I find this article very relevant and interesting.  My wife has been a patient for many years and some years back she was sent to the emergency ward at Haukeland several times.  If the personnel on duty had been trained in the most common diseases that come to the emergency ward and could rapidly have sent them on to the right wards they would have had time to take care of the patients with other symptoms earlier.  In those days, it was normal to wait for up to 5 hours before the duty doctor had time to look at you because there was not a very good system to categorize the patients quickly in order to send the obvious ones to their right wards.  If done as the article says it would eased the burden for both the patients and the personnel working on the Emergency ward.

A first screening on arrival, as it is done to day at the Emergency ward at Haukeland has made the Emergency ward so much more efficient.

As a relative I find this very helpful both for the patients, but also for me as a relative.
